# Parenting and Feeding Styles and Parents’ Body Mass Index as Predictors of Body Mass Index and Disordered Eating Behaviors in Mexican Children

**DOI:** 10.3390/nu17172797

**Published:** 2025-08-28

**Authors:** Iraís Castillo Rangel, Said Jiménez, Claudia Unikel Santoncini

**Affiliations:** 1 Área de Ciencias de la Salud, Unidad Académica de Enfermería, Universidad Autónoma de Zacatecas, Campus UAZ Siglo XXI, Carretera Zacatecas Guadalajara km 6, Colonia La Escondida, Zacatecas 98160, Mexico; irais.castillo@uaz.edu.mx; 2 Escuela de Medicina y Ciencias de la Salud, Tecnologico de Monterrey, Prolongación Canal de Miramontes, Tlalpan, Ciudad de México 14380, Mexico; said.ejp@tec.mx; 3 Dirección de Investigaciones Epidemiológicas y Psicosociales, Instituto Nacional de Psiquiatría Ramón de la Fuente Muñiz, Calzada México-Xochimilco 101, Colonia San Lorenzo Huipulco, Tlalpan, Ciudad de México 14370, Mexico

**Keywords:** parenting styles, feeding styles, BMI, disordered eating behaviors

## Abstract

**Background/Objective:** Problems associated with eating and weight in childhood are complex and have a multifactorial etiology. In recent years, childhood obesity has become a global public health problem with short- and long-term physical, psychological, and social health consequences. This is a cross-sectional study that evaluates the relationship between parenting styles, eating styles, and parents’ body mass index (BMI) and their children’s body mass index (BMI) and disordered eating behaviors (DEBs). **Method:** A sample of 372 dyads of mothers or fathers (mean age = 38 (SD = 8.8)) and boys or girls (mean age = 8.9 years (SD = 0.31)) was used. **Results:** Path analysis found that an authoritarian parenting style had a significant positive relationship with food approach (β = 0.36, *p* < 0.001) and food avoidance factors (β = 0.23, *p* < 0.001). Parental depression was positively associated with food approach (β = 0.20, *p <* 0.001) and food avoidance factors (β = 0.19, *p <* 0.001). Food approach factors significantly predicted compensatory behaviors (β = 0.14, *p <* 0.001). Lastly, both binge eating (β = 0.10, *p <* 0.05) and compensatory behaviors (β = 0.31, *p <* 0.001) showed significant positive relationships with children’s BMI. **Conclusions:** A clearer understanding of the relationships among these factors could facilitate earlier and more effective interventions targeting nutrition- and weight-related issues.

## 1. Introduction

Problems associated with eating and weight in childhood are complex and have a multifactorial etiology. In recent years, childhood obesity has become a global public health problem, with short- and long-term physical, psychological, and social health consequences [1]. In Mexico, the combined prevalence of overweight and obesity in children between 5 and 11 years of age is 38% according to data from ENSANUT 2020 [2]. Although disordered eating behaviors (DEBs) show a higher prevalence in the adolescent population, they are already present in the pediatric population [3]. ENSANUT 2018 reported that, among 10- to 11-year-olds in Mexico, 3.9% were at moderate risk and 0.4% at high risk for DEBs [4]. The prevalence and complexity of these problems raise the need to understand the mechanisms of action of the etiological factors involved to enhance the prevention and treatment of these conditions. The reductionist view that solely attributes overweight and obesity to consuming more calories than one expends ignores predisposing biological factors, early eating practices, emotional factors, geographical location, and familial and social practices [5].

Harrison et al. [6] devised a framework integrating biological, environmental and hereditary factors known as the Six-Cs model (cell, child, clan, community, country, and culture). The latter was based on Bonfenbrenner & Ceci’s model [7] describing the interaction between biological factors and the environment in shaping development. Regarding biological factors, evidence has been found of a genetic predisposition to obesity, and of metabolic processes that determine appetite regulation and the hedonic response to food [8]. This predisposition, however, is expressed in terms of contextual and personal determinants. Davison & Bitch [9] posit that childhood obesity is associated with the context in which a child develops, both individually, within their families, and in the broader community.

Although studies comparing obesity between parents and children are heterogeneous, there is evidence that the body mass index (BMI) of parents and children is related and that children with parents with obesity or overweight are more prone to obesity [10,11]. Meta-analysis studies have found a strong association between obesity in parents and their children. Children with parents with obesity or overweight are more than twice [12] or 1.97 times [13] as likely to have obesity than those with parents with normal weight. Although parents shape their children’s eating behaviors and influence preferences and habits [14], biological factors also play a role [8]. Further investigation is needed to determine the importance of each of these factors.

Family influence plays a critical role in shaping children’s nutritional status. The intergenerational transmission of appetite traits from mothers to children is well-documented [15]. Children’s eating habits partly reflect those of their mothers and immediate environment. In addition to biological predisposition, the correlation may also be due to parental modeling through vicarious learning [16], in which parents with healthy eating behaviors have less of a need to model healthy eating parenting practices, such as avoiding food rewards [17]. Food approach is associated with the entire BMI range, whereas food avoidance traits are negatively associated with weight, protecting against obesity. Conversely, emotional overeating and food responsiveness are associated with higher BMI in both mothers and their offspring [15].

Another key aspect is the parenting style existing within families. Poor family relationships with ineffective communication, high conflict levels, limited behavioral control, and low family hierarchy levels have been found to carry a higher risk of obesity and overweight in children [18,19]. In those with obesity, both a lack of parental interest in children’s eating behavior and excessive control regarding food have been reported [20]. Parents with an authoritative parenting style have children with a lower BMI and their children have healthier diets, whereas parents with an authoritarian parenting style have children with less healthy diets [21]. Although the variables involved in family dynamics in relation to children’s diet and BMI remain unclear, parenting style evidently constitutes a determining factor [22].

The psychopathology of parents has been associated with the psychopathology of their children and may be a predictor of obesity [23,24]. Mothers of children with overweight or obesity have higher indices of psychopathology and maladaptive coping skills than those with children with healthy weight [25]. In children with obesity, with or without a psychiatric diagnosis, statistically significant differences have been found in mothers’ emotional over-involvement and psychological distress in comparison with those of children who are not overweight [26]. Indices of anxiety, overprotection, and criticism are higher in families of children with obesity compared with those of children with healthy weight [25]. When children are raised in an unfavorable family environment, with financial insecurity, a limited ability to express themselves emotionally, or a high level of distress, the emotional burden may encourage DEBs and the use of food as a coping strategy, which is why children may have high levels of emotional eating [27,28,29]. Studies have been conducted on the link between emotional and psychological stress, unfavorable socioeconomic conditions, particularly in non-harmonious family settings, and their role as a source of psychological problems in children, who, lacking effective coping skills, use food to manage conditions such as negative affects, emotional overload, and stress. This, in turn, causes appetite dysregulation, chronic low-grade inflammation, higher levels of emotional eating, and obesity [27]. There is a partial mediation of depression, anxiety, and attitudes related to eating disorders with lack of control when eating and expressed emotion [30].

This study seeks to understand the relationship between parenting style, parental depression levels, and parents’ BMI and their children’s BMI, DEBs, and feeding styles (food approach and food avoidance behaviors). Understanding the relationship between these factors will facilitate the timely, effective treatment of nutrition- and weight-related problems.

## 2. Method

### 2.1. Sample

This study was conducted in the states of Campeche, in southern Mexico, and Mexico City, in central Mexico. There are differences in population size between the two contexts: Campeche has a relatively small population and a slower pace of life, while Mexico City is a much more diverse metropolis. The population in both cities was urban, and the children attended public schools, so their socioeconomic status could be similar.

Three hundred and seventy-two dyads of parents and 4th grader children were analyzed. Participants were recruited using a non-probabilistic convenience sampling method from 16 public primary schools (eight in Campeche and eight in Mexico City) between November 2023 and February 2024. The final dataset included complete data for all participants. The mean age (standard deviation) of the parents was 38 (±8.72) years, and the mean age (SD) of the children was 8.98 (±0.35) years (Table 1).

### 2.2. Instruments

#### Scales Administered to Parents

The Brief Questionnaire on Disordered Eating Behaviors (BQDEB) comprises 10 questions exploring concern about weight gain, feelings of loss of control when eating, restrictive and purging behaviors, and binge eating in the past three months. The Questionnaire has four Likert-type response options: never or almost never, sometimes, frequently (2 times a week), and very frequently (more than 2 times a week), in the three months prior to the assessment. The questionnaire was validated in adolescents and adults in clinical and community populations. The factorial structure of the questionnaire was organized into three factors explaining 64.7% of the variance with a Cronbach’s alpha of 0.76 [31].

Center for Epidemiologic Studies Depression Scale (CES-D) [32]: The Center for Epidemiologic Studies Depression Scale (CES-D) comprises 20 questions measuring depression symptoms. Participants were asked to report the number of days they had experienced each of the symptoms during the previous week (0, 1–2, 3–4, 5–7) [32]. The validation of the scale conducted in Mexico [33] with 873 middle and high schoolers demonstrated that the scale has a high level of reliability (Cronbach’s alpha = 0.88) and a well-defined factor structure indicating the existence of three subscales: negative affect, somatic symptoms, and positive affect. For the present sample, alpha was 0.88, 95% CI [0.87, 0.90], and omega was 0.92.

Parenting Styles: The Parenting Practices Questionnaire, developed by Robinson et al. [34], is based on Baumrind’s [35,36,37] typology of parenting styles. Consisting of 62 questions administered to 1251 parents with preschool and elementary school children [38], it is scored on a Likert scale with five response options (never, sometimes, about half the time, very often, and always). The authors reported significant Cronbach’s alphas for the scales used: 0.91 for the 27 items associated with the authoritative scale, 0.86 for the 20 questions on the authoritarian scale, and 0.75 for the 15 questions on the permissive scale. The research team for this study conducted a validation study of a sample of 272 mothers, with ages ranging from 26 to 52 years (M = 39.16, SD = 5.56) with children in elementary school at two private schools in Mexico City. The children, aged between 6 and 13 (M = 8.77, SD = 1.85), were enrolled in the following grades: first (18%), second (16.5%), third (15.4%), fourth (15.4%), fifth (15.1%), and sixth (19.5%). Due to the number of items in the questionnaire and the fact that the sample size was not sufficient to conduct an exploratory factor analysis with all the items, it was decided to conduct a second-order factor analysis with the 11 initial factors. As a result, the analysis yielded two dimensions: authoritarian and authoritative parenting. The total explained variance was 58.14%, with a reliability of 0.79. This instrument, comprising 39 questions and two dimensions, was used in the present research. For the sample in this study, the authoritarian parenting dimension obtained an alpha of 0.88, 95% CI [0.86, 0.89], and an omega of 0.89, while the authoritative parenting dimension resulted in an alpha of 0.76, 95% CI [0.73, 0.79], and an omega of 0.80.

Child Eating Behavior Questionnaire (CEBQ). This questionnaire comprises 35 questions with five response options in a Likert format (1 = never, 2 = rarely, 3 = sometimes, 4 = often, and 5 = always). The CEBQ measures four food approach subscales: Food Responsiveness, Emotional Over-eating, Enjoyment of Food, and Desire to Drink. It also measures four food avoidance subscales: Satiety Responsiveness, Emotional Under-eating, Food Fussiness, and Slowness in Eating. It has a Cronbach’s alpha reliability between 0.68 and 0.86 and a McDonald’s omega between 0.71 and 0.87. Confirmatory factor analysis of the original factor structure of the CEBQ showed a valid structure of eight subscales with an adequate fit to the model [39]. In the present study, only two dimensions were used in the analysis: food approach and food avoidance.

### 2.3. Scales Administered to Girls and Boys

Brief Questionnaire for Measuring Children’s Disordered Eating Behaviors. The questionnaire consists of seven questions scored on a three-choice response scale (No = 0, Sometimes = 1, Yes = 2). It is organized into two scales: (1) questions on binge eating and lack of control when eating, with an ordinal alpha of 0.90, and (2) questions on compensatory behaviors or food restriction, with an ordinal alpha of 0.90. The overall reliability of the scale is 0.91. Confirmatory factor analysis showed adequate fit indices to the model [40].

Self-Administered Children’s Binge Eating Disorder Scale (SA-C-BEDS): This scale consists of five questions with two response options: yes = 1 and no = 0. A higher score on the scale indicates more binge eating behavior. It was validated in Mexican children with an average age of 10.22 years (SD = 0.94). Factor analysis showed that the scale is distributed in a single factor, with a significant Bartlett’s test of sphericity, 0.73 in the Kaiser–Meyer–Olkin test, and an ordinal alpha reliability of 0.81. Confirmatory validity showed that the model fits the data and is valid [41].

### 2.4. Body Mass Index (BMI)

Parental BMI was calculated by obtaining direct measurements of the weight in kilograms and height in centimeters of each participant. The cut-off points proposed by the WHO [42] for adults were used: <18.5 = underweight, 18.5–24.9 = normal weight, 25–29.9 = overweight, and ≥30 = obesity.

Children’s weight and height measurements were obtained by trained personnel using standardized procedures. The following steps were taken to calculate the Z score of the BMI: (1) to determine a person’s BMI (weight [kg]/height^2^ [m^2^]); (2) consult the World Health Organization (WHO) reference values for age and sex, specifically the median (P50) and standard deviation (SD) of the population distribution; and (3) apply the Z = (observed BMI—reference median BMI)/reference SD formula). This value quantifies standard deviations from the median, allowing the classification of nutritional status according to predefined thresholds (for example Z < −2: underweight; Z > 1: risk of overweight; Z > 2: obesity). The Anthro/AnthroPlus package version 3.2.2 [42,43,44,45] was used for this calculation.

### 2.5. Procedure

To administer the questionnaires, a capture mask was designed using the Census and Survey Processing System (CSPRO) program, structured around dictionaries (tables), forms, and the programming of various variables for data collection. Parents were invited to the schools on a specific day to be assessed by trained personnel, including weight and height measurements and psychosocial questionnaires.

For the anthropometry measurement, we used Seca 874 electronic floor scales (Manufacturer: Seca GmbH, Hamburg, Germany) with a 200 kg capacity and accuracy levels of 50 g below 100 kg and ±100 g above 200 kg, a 205 cm-high Seca 213 portable stadiometer (Manufacturer: Seca GmbH, Hamburg, Germany) with 1 mm accuracy and a 2 m-long Seca fiberglass tape measure with 1 mm accuracy.

### 2.6. Data Analysis

A path analysis model was used to evaluate the set of multivariate relationships predicting the BMI in children. This allows for the empirical examination of a chain of relationships that begins with the characteristics of the parenting style and parental depression levels and continues with food approach and food avoidance factors in children, as well as their DEBs, and culminates in the BMI.

We explored a model positing that parental depression, together with authoritarian and authoritative parenting styles, predict children’s food approach and food avoidance factors. Both parental depression and an authoritarian parenting style were expected to have positive relationships with these factors (such as greater emotional overeating or undereating), whereas an authoritative style was expected to be a negative predictor. At the same time, the food approach and food avoidance factors were proposed as predictors of DEBs, including both binge eating and compensatory behaviors. For example, emotional overeating was expected to increase the likelihood of binge eating and compensatory behaviors, whereas emotional undereating would be associated with a lower occurrence of these behaviors. Finally, it was hypothesized that both binge eating and compensatory behaviors would be positively associated with BMI.

To test this hypothesized model of direct relationships between observed variables, two path analyses were fitted and compared using the *lavaan* package [46] in R 4. 5. 1. The first analysis assessed the model described above, whereas the second analysis added the control variables: sex, age of the child, and BMI of the parent. Except for the sex of the child, which was binary-coded, the other variables were assumed to be continuous and directly observed, and therefore the maximum likelihood (ML) estimator was used. The model fit was evaluated using the χ^2^, CFI, TLI, RMSEA, and SRMR indices, following the criteria proposed by Hu and Bentler [47].

### 2.7. Ethical Considerations

All project procedures, questionnaires, and consent and assent letters were reviewed and approved by the Ethics and Research Committees of the National Institute of Public Health (protocol code 1542 received on 13 April 2021). All parents were asked to sign informed consent forms for their own participation and that of their offspring. The children were also asked to provide their informed consent.

## 3. Results

### 3.1. Model Comparison

Two path models were compared: one without covariates and another including sex, age, and parental BMI as covariates. Both models showed an acceptable overall fit and comparable magnitude. The model without covariates showed a fit of χ^2^ (13) = 25.16, *p =* 0.02, CFI = 0.94, TLI = 0.89, RMSEA = 0.05, SRMR = 0.04, AIC = 10,045, and BIC = 10111. Conversely, the model with covariates had a fit of χ^2^ (13) = 26.54, *p =* 0.01, CFI = 0.95, TLI = 0.83, RMSEA = 0.05, SRMR = 0.03, AIC = 10,025, and BIC = 10,150.

Because the models are not nested (in other words, the model with covariates incorporates additional variables not found in the first one), the overall fit indices are not directly comparable. However, it was decided to report the standardized results of the model with covariates, since sex, age, and parental BMI are plausible confounders, with possible effects on both the predictors and dependent variables. The standardized coefficients of both models, which were very similar, can be seen in Table 2. Descriptive statistics for all variables included in the path models are presented in Supplementary Materials: Table S1.

### 3.2. Model Results with Covariates

**Figure 1:**
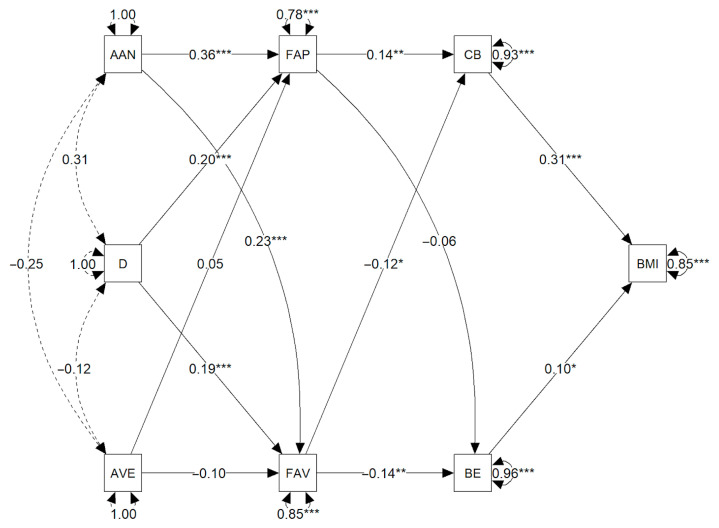
Path model of parenting styles, feeding styles, DEBs, and child body mass index. Standardized coefficients are given, showing the direction of the hypothesized paths, and magnitude and level of statistical significance. One-way arrows indicate effects assessed via regression, whereas double-headed arrows reflect correlations if they involve two variables and variances if they involve a single variable. FAP = food approach, FAV = food avoidance, CB = compensatory behaviors, BE = binge eating, AVE = authoritative, AAN = authoritarian, D = depression, BMI = body mass index. “*” *p <* 0.05; “**” *p <* 0.01; “***” *p <* 0.001.

Path modeling with covariates empirically supported the exploratory hypothesized chain of relationships, even when controlling for parental sex, age, and BMI (Figure 1). Results revealed that an authoritarian parenting style had significant positive relationship with food approach (β = 0.36, SE = 0.05, *p <* 0.001) and food avoidance (β = 0.23, SE = 0.05, *p <* 0.001) factors. Likewise, parental depression was positively associated with food approach (β = 0.20, SE = 0.05, *p <* 0.001) and food avoidance (β = 0.19, SE = 0.05, *p <* 0.001) factors. Contrary to expectations, an authoritative parenting style failed to show significant effects on either food approach (β = 0.05, SE = 0.05, *p =* 0.30) or food avoidance factors (β = −0.10, SE = 0.05, *p =* 0.05).

Regarding DEBs, food approach factors significantly predicted compensatory behaviors (β = 0.14, SE = 0.05, *p <* 0.001), yet did not show a significant association with binge eating (β = −0.06, SE = 0.05, *p =* 0.25). At the same time, food avoidance factors were negatively associated with both compensatory behaviors (β = −0.12, SE = 0.05, *p <* 0.001) and binge eating (β = −0.14, SE = 0.05, *p < 0.05*). Finally, both binge eating (β = 0.10, SE = 0.05, *p <* 0.05) and compensatory behaviors (β = 0.31, SE = 0.05, *p <* 0.001) showed positive significant predictions of children’s BMI (Figure 1).

## 4. Discussion

The objective of this study is to understand the relationship between parenting styles, parental depression levels, and parents’ BMI and their children’s BMI, DEBs, and food approach and food avoidance behaviors.

Parenting styles may modify eating patterns, encouraging either food approach or food avoidance, or encourage balanced eating behavior. In the present study, the authoritarian parenting style variable had significant positive relationships with children’s food approach and food avoidance factors, unlike the authoritative parenting style, which showed no relationships with these variables. That is to say, the authoritative parenting style has no significant association with the variables of food approach or food avoidance, which indicates a healthier relationship with food. This in turn would not have an impact on DEBs and would be a protective factor for problems related to food and weight.

Our results are consistent with those of previous studies. For example, in a meta-analysis [48], Pinquart (2013) [48] found that the general parenting style (referring to the emotional climate of parenting practices) and positive parent–child relationships with a higher level of responsiveness were associated with lower BMI, healthier eating, and more physical activity in children.

In a systematic review, Sleddens et al. [49] found that the authoritative parenting style is also associated with lower BMI, healthier eating, and more physical activity in children, as did Berge’s [22] study. Along these same lines, in a review of five studies, Skouteries et al. [50] found that the interaction between parents and children is associated with the regulation of children’s weight. Although inadequate parental support, poor communication, and low levels of involvement were associated with higher BMI, one of the five studies reviewed found the opposite. The explanation is that mothers who participated in the study and used mealtimes to interact with their children may have had a higher BMI and modeled an association between food and positive emotions.

Both the literature reviewed and the results found in the present study demonstrate the importance of parenting style in children’s BMI. The mechanism through which parenting style directly influences children’s eating and weight problems has several possible explanations which have part of their origin in the parenting style. Self-regulation as a construct involving behavioral, cognitive, physiological, and emotional aspects appears to play a key role in the regulation of eating and weight [51,52].

A parent–child relationship in which there is mutually responsive orientation, routines, and communication between parents and their children, and occurs without great effort or conflict and in a favorable emotional environment, can contribute to overall self-regulation in children and therefore better self-regulation in food intake, protecting against overweight and obesity [50]. Graziano et al. [53] found that children as young as two years old with better self-regulation were less likely to present pediatric obesity, and that children with greater adiposity have lower levels of self-regulation [54].

Conversely, the authoritative parenting style promotes higher levels of self-regulation in children in general [50,51], including self-regulation of body weight. In regard to food, an authoritative parenting style sets limits on the way children eat while giving them autonomy [55]. It has been shown that a structured, more authoritative, and less permissive parenting style encourages children to have a lower behavioral response to external food stimuli, such as restaurant logos or advertisements. Children with lower emotional self-regulation, inhibitory control, and attentional focus were associated with having a greater response to these stimuli [56].

Low inhibitory control was significantly associated with greater appetitive traits, which may reflect a neurobiological drive to consume food [56]. Low emotional regulation and response to food cues have been proposed as risk factors for childhood obesity [51], while the authoritative parenting style protects children from eating disorders.

In contrast to the authoritative parenting style, which promotes better regulation of eating behavior, in the present study, the authoritarian parenting style variable was significantly related to food approach and food avoidance behaviors. In turn, food approach behavior predicted compensatory behaviors, although not binge eating. There is evidence that the specific pattern of Binge Eating Disorder is characterized by increased impulsivity both in general and specifically towards food [57] so it could be suggested that food approach behavior predicts compensatory behaviors but binge eating behavior would be mediated by a high component of impulsivity.

Authoritarian parents often restrict or require certain actions without explanation, including controlling children’s food choices or removing food they enjoy. When parents restrict their children’s consumption of foods high in fat and sugar, which is a type of authoritarian control, children crave these forbidden foods and tend to eat them even when they are full [58]. This restriction has been associated with obesogenic behaviors in children since it increases the desire to consume these foods [51].

When feeding practices have been evaluated more specifically, studies have found that authoritarian parenting is associated with lower vegetable and dairy consumption, regardless of ethnicity, socioeconomic status, or education [55]. Another study found that, when parents restrict highly palatable foods in their daughters, it generates negative self-evaluation regarding their consumption of these foods. However, this does not cause them to consume less of them. Indeed, it can paradoxically promote the consumption of these foods while generating negative feelings towards their consumption [58].

DEBs as unhealthy weight control methods have been linked to detrimental parenting styles, characterized by high levels of control and low levels of responsiveness [59]. Parenting styles at either extreme such as authoritarian and neglectful styles precipitate cascading behaviors that can lead to eating disorders [60], while authoritarian parenting styles, with avoidant and over-compensatory characteristics, are related to problematic eating attitudes and behaviors [61].

In the model obtained, parental depression was positively associated with food approach and food avoidance factors, and food approach behavior ultimately predicted a higher BMI. Based on a systematic review, Marco et al. [62] concluded that there is a positive relationship between parental anxiety and depression symptoms and children’s obesity, with our study finding similar results. Other studies have also shown that maternal depression symptoms are associated with children with higher weight [63]. These mothers exert greater verbal pressure on children to eat during mealtimes and are less likely to encourage them to be independent [64].

The degree of depression has also been shown to be important. Mothers with higher levels of depression displayed less responsive feeding practices than mothers with lower levels of depression [65]. In turn, maternal responsiveness is necessary for optimal child development, including the capacity for self-regulation [66], which, as mentioned, plays a determining role in eating behavior.

Conversely, some studies have associated maternal depression with children with low weight [67]. These results agree with the association between depression and food avoidance patterns found in the present study. Other models explaining how the family environment determines the BMI of children include one proposed by Hemmingson [27], stating that, when children grow up in an unharmonious family environment, they are exposed to parental frustration and lack of support and cohesion, resulting in unmet emotional needs and insecurity. Although the central feature of Hemmingson’s model is socioeconomic disadvantage, a variable we did not consider, we can hypothesize that parental depression and authoritarian parenting styles create a less supportive family environment for children, and that emotional needs are less met. Hemmingson posits that these familial characteristics increase psychological and emotional distress in children, creating psycho-emotional overload that triggers a cascade of maladaptive behaviors such as eating to suppress negative emotions, chronic stress, and poor appetite regulation. 

The results found in this study present the chain of relationships and the potential impact that parenting style, depression, and parental BMI have on eating disorders in children as an etiological factor. The food approach eating pattern triggers DEBs, which, in addition to predisposing children to suffering from a future eating disorder, might affect their BMI.

Parenting style and parental mental health have been shown to be key factors in both the prevention and management of eating and weight-related problems. The findings can be integrated into the content of preventive programs for eating disorders and obesity aimed at parents and teachers. It is also essential that healthcare personnel understand the impact of these factors to provide more comprehensive care for problems related to eating disorders and obesity. It is important for both families and professionals involved in addressing these issues to consider the protective role that an authoritative parenting style plays in children’s overall health and for these aspects to be included in interventions.

## 5. Limitations of the Study

Limitations of the study include the fact that, as it is a cross-sectional study, we do not know how these factors could evolve in the medium and long term, and although it may be implied, it is necessary to conduct more research to prove causality. For example, longitudinal studies are needed to better understand the impact of parenting style, depression, and parental BMI on children’s eating habits. It would also be advisable to conduct a study in which, in addition to collecting data, parenting practices could be directly observed.

The questionnaires may be affected by self-reporting bias such as social desirability, memory bias, and unawareness of their own behaviors. Besides this, another potential limitation is the use of self-report instruments in young children (8–9 years), as memory biases or lack of awareness of one’s own behaviors may be greater. Social desirability scales, which would have shed more light on this issue, were not used; however, the instruments used in this study have been adequately validated in this population to minimize potential biases.

As for data generalizability, there are several points worth mentioning. First, since the sample consisted of a higher percentage of mother–child dyads, and father–child dyads were underrepresented, the results mainly show the influence of parenting practices and feeding styles of mothers rather than fathers. At the same time, the age range of the sample of children is small and does not represent what may happen with the variables studied in other age groups. This study was conducted in just two states in Mexico (Campeche and Mexico City), and Mexico is a diverse country, with significant cultural differences by region.

The parenting practices questionnaire was reduced to just two dimensions during its validation, meaning that key aspects of parenting styles may be overlooked. Finally, there may be other confounding factors besides those included in the analysis, such as socioeconomic status, parental education level, access to healthy food, physical activity, genetic predispositions, and specific eating practices.

## 6. Conclusions

The results of this study show the association between parenting style, and parents’ depression levels and BMI, and their children’s BMI, DEBs, and food approach and food avoidance behaviors. Both depression and an authoritarian parenting style lead to altered eating patterns, both food approach and food avoidance, unlike the authoritative parenting style, which was not associated with either of these behaviors.

Both variables have a high level of correlation with food approach behavior, which in turn is related to compensatory behaviors, ultimately resulting in a higher BMI. The mechanism by which these variables alter eating behavior could be due to factors such as self-regulation, emotional management, family environment, level of parental support, and level of responsiveness and control by parents toward their children.

Both parenting style and parental depression have been shown to be key factors in the etiology of problems related to children’s eating behavior and BMI. These psychosocial variables must be considered in both the prevention and management of problems related to children’s eating and weight.

## Figures and Tables

**Table 1:** Demographic and anthropometric characteristics of parent–child dyads.

N = 372	Mean	SD
Child age in years (mean (SD))	8.98	0.35
Parent age in years (mean (SD))	38.02	8.72
Parent body mass index	28.20	4.40
	Frequency	%
Child sex		
Female	194	52.2
Male	178	47.8
Parent sex		
Female	332	89.2
Male	40	10.8
Parent’s educational attainment		
Elementary school	109	29.3
High school	152	40.9
College and graduate levels	111	29.8
Child body mass index (%)		
Underweight	4	1.1
Healthy weight	185	49.7
Overweight	76	20.4
Obesity	107	28.8

**Table 2:** Results of path analysis with standardized estimates and 95% confidence intervals (CIs).

Model	Outcome	Predictor	Estimate	CI (2.5%)	CI (97.5%)	SE	Z	*p*
1	BMI	BE	0.12	0.02	0.21	0.05	2.38	0.02
		CB	0.3	0.21	0.39	0.05	6.42	<0.001
	BE	FAP	−0.07	−0.17	0.03	0.05	−1.42	0.16
		FAV	−0.14	−0.24	−0.04	0.05	−2.65	0.01
	CB	FAP	0.17	0.07	0.26	0.05	3.25	<0.001
		FAV	−0.15	−0.25	−0.05	0.05	−2.9	<0.001
	FAP	AVE	0.05	−0.04	0.14	0.05	1.06	0.29
		AAN	0.37	0.28	0.46	0.05	8.17	<0.001
		D	0.2	0.11	0.3	0.05	4.32	<0.001
	FAV	AVE	−0.1	−0.2	0	0.05	−2.04	0.04
		AAN	0.22	0.12	0.32	0.05	4.28	<0.001
		D	0.19	0.09	0.28	0.05	3.72	<0.001
2	BMI	BE	0.1	0.01	0.2	0.05	2.16	0.03
		CB	0.31	0.22	0.4	0.05	6.58	<0.001
		Girl	0	−0.09	0.1	0.05	0.1	0.92
		Age	0	−0.1	0.09	0.05	−0.09	0.93
		BMI-P	0.22	0.12	0.31	0.05	4.6	<0.001
	BE	FAP	−0.06	−0.16	0.04	0.05	−1.16	0.25
		FAV	−0.14	−0.25	−0.04	0.05	−2.81	<0.001
		Girl	0.08	−0.03	0.18	0.05	1.47	0.14
		Age	0.06	−0.04	0.16	0.05	1.09	0.28
		BMI-P	0.08	−0.02	0.18	0.05	1.5	0.13
	CB	FAP	0.14	0.04	0.24	0.05	2.78	0.01
		FAV	−0.12	−0.22	−0.02	0.05	−2.41	0.02
		Girl	−0.18	−0.28	−0.08	0.05	−3.59	<0.001
		Age	0.01	−0.08	0.11	0.05	0.29	0.77
		BMI-P	−0.05	−0.15	0.05	0.05	−0.99	0.32
	FAP	AVE	0.05	−0.04	0.14	0.05	1.03	0.3
		AAN	0.36	0.27	0.45	0.05	7.92	<0.001
		D	0.2	0.11	0.3	0.05	4.33	<0.001
		Girl	−0.08	−0.17	0.01	0.05	−1.76	0.08
		Age	−0.03	−0.12	0.06	0.05	−0.63	0.53
		BMI-P	−0.02	−0.11	0.08	0.05	−0.34	0.73
	FAV	AVE	−0.1	−0.19	0	0.05	−1.94	0.05
		AAN	0.23	0.13	0.32	0.05	4.5	<0.001
		D	0.19	0.09	0.28	0.05	3.79	<0.001
		Girl	0.14	0.04	0.23	0.05	2.87	<0.001
		Age	−0.06	−0.15	0.03	0.05	−1.25	0.21
		BMI-P	0.04	−0.05	0.14	0.05	0.84	0.4

FAP = food approach, FAV = food avoidance, CB = compensatory behaviors, BE = binge eating, AVE = authoritative, AAN = authoritarian, D = depression, BMI-P = parental.

## Data Availability

The datasets generated during and/or analyzed during the current study are available from the corresponding author on reasonable request.

## Supplementary Materials

The following supporting information can be downloaded at: https://www.mdpi.com/article/10.3390/nu17172797/s1, Table S1: Summary statistics for variables in the path models
