# Supplementary material for: Parenting and Feeding Styles and Parents’ Body Mass Index as Predictors of Body Mass Index and Disordered Eating Behaviors in Mexican Children
Source: Nutrients. 2025 Aug 28;17(17):2797. doi: 10.3390/nu17172797 (PMC12430279; doi:10.3390/nu17172797)
Supplement: Supplementary file 1 [file nutrients-17-02797-s001.zip › nutrients-3783513-supplementary.pdf]

Supplementary table

Variable	N	M	SD	MIN	MAX	Skewness	Kurtosis
Depression	372	35.49	8.13	20	73	0.99	1.86
Authoritarian	372	33.3	7.06	21	59	0.71	0.58
Authoritative	372	76.66	11.91	38	95	-0.87	0.45
Proingest	372	40.62	10.12	16	77	0.88	0.7
Antiingest	372	47.74	9.41	19	78	0.22	0.33
Binge	372	2.01	1.64	0	6	0.49	-0.65
Compensatory	372	2.09	1.89	0	8	0.72	-0.08
BMI	372	18.84	3.53	12.33	27.35	0.49	-0.72
BMI parent	372	28.2	4.4	18.79	39.8	0.31	-0.37
